# Potential of ChatGPT in youth mental health emergency triage: Comparative analysis with clinicians

**DOI:** 10.1002/pcn5.70159

**Published:** 2025-07-15

**Authors:** Samanvith Thotapalli, Musa Yilanli, Ian McKay, William Leever, Eric Youngstrom, Karah Harvey‐Nuckles, Kimberly Lowder, Steffanie Schweitzer, Erin Sunderland, Daniel I. Jackson, Emre Sezgin

**Affiliations:** ^1^ Department of Psychiatry and Behavioral Health The Ohio State University Columbus Ohio USA; ^2^ Nationwide Children's Hospital Columbus Ohio USA; ^3^ The Abigail Wexner Research Institute, Nationwide Children's Hospital Columbus Ohio USA

**Keywords:** artificial intelligence, emergency, mental health, suicide, triage

## Abstract

**Aim:**

Large language models, such as GPT‐4, are increasingly integrated into healthcare to support clinicians in making informed decisions. Given ChatGPT's potential, it is necessary to explore such applications as a support tool, particularly within mental health telephone triage services. This study evaluates whether GPT Models can accurately triage psychiatric emergency vignettes and compares its performance to that of clinicians.

**Methods:**

A cross‐sectional study was performed to assess the performance of three different GPT‐4 models (GPT‐4o, GPT‐4o Mini, and GPT‐4 Legacy) in psychiatric emergency triage. Twenty‐two psychiatric emergency vignettes, intended to represent realistic prehospital triage scenarios, were initially drafted using ChatGPT and subsequently reviewed and refined by the research team to ensure clinical accuracy and relevance. The GPT‐4 models independently generated clinical responses to the vignettes over three iterations to ensure consistency. Thereafter, two advanced practice nurse practitioners independently assessed these responses utilizing a 3‐point Likert‐type scale for the main triage criteria: risk level (*Low* = 1 to *High* = 3), necessity of hospital admission (*Ye*s = 1; *No* = 2), and urgency of clinical evaluation (*Low* = 1 to *High* = 3). Additionally, the nurse practitioners provided their clinical judgments independently for each vignette. Interrater reliability was evaluated by comparing responses generated by the GPT Models with the independent clinical assessments of nurse practitioners, and agreement was evaluated using Cohen's Kappa. The clinical expert committee (*n* = 3) conducted qualitative analyses of responses from both GPT Models using a systematic coding method to evaluate triage accuracy, clarity, completeness, and total score. The evaluation of responses focused on three key triage criteria: risk (*Low* = 1 to *High* = 3), admission necessity (*Ye*s = 1; *No* = 2), and urgency of clinical evaluation (*Low* = 1 to *High* = 3).

**Results:**

GPT Models had an average admission score of 1.73 (standard deviation [SD] = 0.45; scale: *Yes* = 1, *No* = 2), indicating a general trend toward recommending against hospital admission. Risk (mean = 2.12, SD = 0.83) and urgency (mean = 2.27, SD = 0.44) assessments suggested moderate‐to‐high perceived risk and urgency (scale: *Low* = 1, *High* = 3), reflecting conservative decision‐making. Interrater reliability between clinicians and GPT‐4 models was substantial, with Cohen's Kappa values of 0.77 (admission), 0.78 (risk), and 0.76 (urgency). GPT Models’ responses tended toward slight over‐triage, indicated by four false‐positive admission recommendations and zero false negatives. Substantial interrater reliability was observed between clinicians and GPT‐4 responses across the three triage criteria (Cohen's Kappa: admission = 0.77; risk = 0.78; urgency = 0.76).The mean scores for triage criteria responses between GPT‐4 models and clinicians exhibited consistent patterns with minimal variability. Overall, GPT Models had a tendency to over‐triage patients as indicated by four total false positives and zero false negatives for admissions.

**Conclusion:**

This study indicates that GPT Models may serve as supportive decision‐support tools in mental health telephone triage, particularly for psychiatric emergencies. Although response variability across iterations was minimal, most discrepancies in admission decisions were identified as false positives, reflecting that GPT Models may have a tendency to over‐triage relative to clinician judgment. Further investigation is needed to establish robust structure to increase alignment with clinical decisions and response relevance in clinical practice.

## Graphical abstract

GPT Models were compared to clinicians in their ability to evaluate 22 emergency psychiatry clinical vignettes. The models exhibited substantial interrater reliability with clinicians along with consistent triage mean scores in admission, urgency, and risk. The findings in our study are promising, but further research is required to confirm the clinical relevance of these GPT Models.

## INTRODUCTION

Young adults and adolescents are currently experiencing a mental health crisis.^1^, ^2^, ^3^ One of the cornerstones of this crisis is a heightened level of suicide risk due to various factors, such as social isolation, hopelessness, and depression.^4^, ^5^ As a result, suicide is the leading cause of death for 10–24‐year‐olds in the United States and requires immediate attention.^6^ Youth presenting with psychiatric emergency conditions can include suicide, assaultive or violent behavior, and acute mental status changes, such as psychosis, intoxication, and extreme anxiety.^7^ For youth experiencing psychiatric emergencies, emergency departments (EDs) are safe areas to visit since they are required to treat all patients without obligation. Approximately half a million children visit the ED every year with urgent psychiatric emergencies^8^; however, EDs often lack the resources to identify and manage patients in psychiatric populations.

Since visiting the ED is not always the most optimal solution for patients with acute psychiatric conditions, prehospital triage helps patients speak with a clinician to determine their best course of action. Prehospital triage is a process in which patients are prioritized by the severity of their condition and their expected resource needs, which are tied to the urgency of their condition.^9^ Mental health prehospital triage is a form of prehospital triage that concentrates more on the risk of harm to self or others in addition to the urgency of the situation. In mental health triage, rapidly and accurately assessing clinical characteristics to determine the severity of psychiatric emergencies is one of the most critical initial steps to providing appropriate treatment.^10^ Mental health emergency hotlines, such as the 988 Suicide and Crisis Lifeline, act as prehospital triage centers and play a vital role in managing patients with acute psychiatric conditions.^11^ Trained responders on these lines can provide a variety of services, such as giving emotional support, providing suicide risk assessments, giving referrals to treatment and even transferring patients to emergency services.^12^ Mental health telephone triage (MHTT) requires clinicians to have competence in resource and time management, working knowledge of psychopharmacology, and knowledge of community‐based services and referrals, and therapeutic approaches and interventions, along with many other skills.^13^ As a result, clinicians cannot always provide consistent and accurate triage responses, which can prevent patients with psychiatric emergencies from receiving effective treatment in a timely manner. Fortunately, there are clinician support tools that can ease the burden on clinicians, such as knowledge‐based decisional algorithms and data‐driven artificial intelligence (AI) models.^14^

AI has been evident in being able to assist clinicians in triaging acute emergency conditions in other specialties.^15^, ^16^ Similarly, prehospital triage models assisted by machine learning, a subset of AI, were shown to have the ability to triage more effectively than conventional models.^17^ AI has also shown promise in being a support tool in online mental health care in the form of chatbots providing immediate mental health support, such as coping strategies, access to mental health professionals, information about mental health conditions, and treatments through a conversational interface.^18^ Additionally, AI models have been shown to help clinicians create better therapeutic applications for psychiatric disorders, such as augmenting traditional therapy by incorporating cognitive behavioral exercises through digital platforms and automated assessment techniques, which improve clinical diagnosis.^19^, ^20^

Despite these advancements, there are still many challenges and limitations to AI‐based mental health services. One drawback is the way in which AI models generate outputs without transparency on how decisions are made—the “black box” problem. As a result, clinicians are unable to identify various biases that are coded into these decisions leading to an inability to minimize bias in clinical decisions in mental health care.^21^ For instance, a recent review highlighted concerns about detrimental aspects of search engine algorithms in suicide prevention efforts,^22^, ^23^ further exemplifying the black box problem. Following this, AI has also shown a lack of ability to consistently provide reliable and accurate clinical information in mental health care. Previous studies have also highlighted concern in the reliability and accuracy of AI models, such as ChatGPT, in making clinical mental health decisions.^24^, ^25^ Other drawbacks include ethical concerns regarding patient autonomy, privacy, and potential stigmatization of patients.^26^

While AI in mental health care does present challenges and limitations, its application in mental health is still in its infancy^27^ and, based on our knowledge of the current literature, there are limited studies observing the ability of AI to support psychiatric care in real‐world settings. A significant area of interest is the potential role of large language models (LLMs) in psychiatric care. LLMs are a specialized category of machine learning models involved in natural language processing and deep learning. Unlike traditional machine learning models, LLMs excel at processing unstructured text^28^ making them valuable in contexts such as MHTT prehospital triage. Although this study primarily focuses on LLMs’ ability to synthesize unstructured text, LLMs’ capacity to synthesize natural language input provides a novel approach to assessing an individual's mental health in mental health settings.^29^ The integration of LLMs into mental health also requires attention to ethical design, cultural nuance, and digital inclusivity to ensure that their benefits are distributed equitably.^30^

The primary aim of this study is to compare the ability of one of the prominent LLMs, GPT‐4 by OpenAI, to triage young adult patients with psychiatric emergencies to that of clinicians based on standardized pre‐prepared clinical vignettes created to simulate real‐world prehospital telephone triage scenarios. A secondary aim of the study is to determine which GPT Model, GPT‐4o, GPT‐4o Mini, or GPT‐4 Legacy, is the most effective in being able to triage patients.

## METHODS

**Figure 1:**
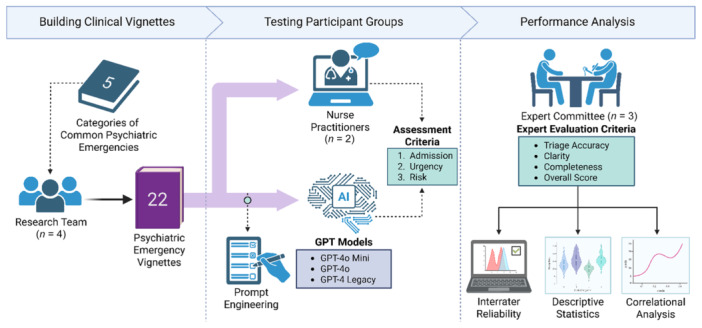
Study design, analysis, and procedures.

In this study, we investigated the ability of GPT‐4 Models via ChatGPT to triage patients with psychiatric emergencies based on risk, admission, and urgency. Figure 1 outlines the study procedure.

### Model selection

GPT‐4 was the LLM selected for this study due to its easy accessibility and its availability to the public. We decided to test the three different GPT‐4 models: GPT‐4o Mini (released on 07/2024), GPT‐4o (released on 05/2024), and GPT‐4 Legacy (released on 03/2023) since they were the most up‐to‐date GPT Models when this study was conducted^31^ (August–September 2024). Out of the three models, GPT‐4 Legacy was released first and is a large multimodal model that accepts image and text inputs with enhanced capabilities in advanced reasoning, complex instructions, and creativity.^32^ GPT‐4o provides GPT‐4‐level intelligence but is much faster and has improved capabilities in text, voice, and vision.^33^ At the time of this study, GPT‐4o is the current state‐of‐the‐art model from OpenAI for public use. GPT‐4o mini was released subsequently after GPT‐4o and is the most cost‐efficient model.^34^ This allowed us to compare performance in both high‐resource and low‐resource deployment scenarios, ensuring relevance for real‐world cases. We will refer to these three models as “GPT Models” from this point forward.

### Question creation

To test different psychiatric emergencies, authors used ChatGPT to simulate a list of 22 clinical vignettes (see Appendix A) across five categories of common psychiatric emergencies: (1) psychosis, (2) suicidal ideation, (3) substance abuse, (4) extreme anxiety, and (5) violent/destructive behavior.^7^ Each vignette template had a paragraph format including information about age, race, gender, medication history, behavioral symptoms, and patient history. The vignettes were initially developed using ChatGPT to create realistic prehospital triage scenarios, simulating calls from caregivers of young adults experiencing mental health crises. Each vignette was initially drafted based on characteristics from one of five predefined categories of mental health psychiatric emergencies. These AI‐generated vignettes were subsequently reviewed for clinical accuracy and utility by S.T., M.Y., I.K., and W.L. Feedback from these clinicians was used to refine and finalize the scenarios, ensuring relevance and clinical validity. One vignette (Number 13) was excluded due to significant disagreement among the clinical reviewers regarding its content and presentation.

### Data collection

**Figure 2:**
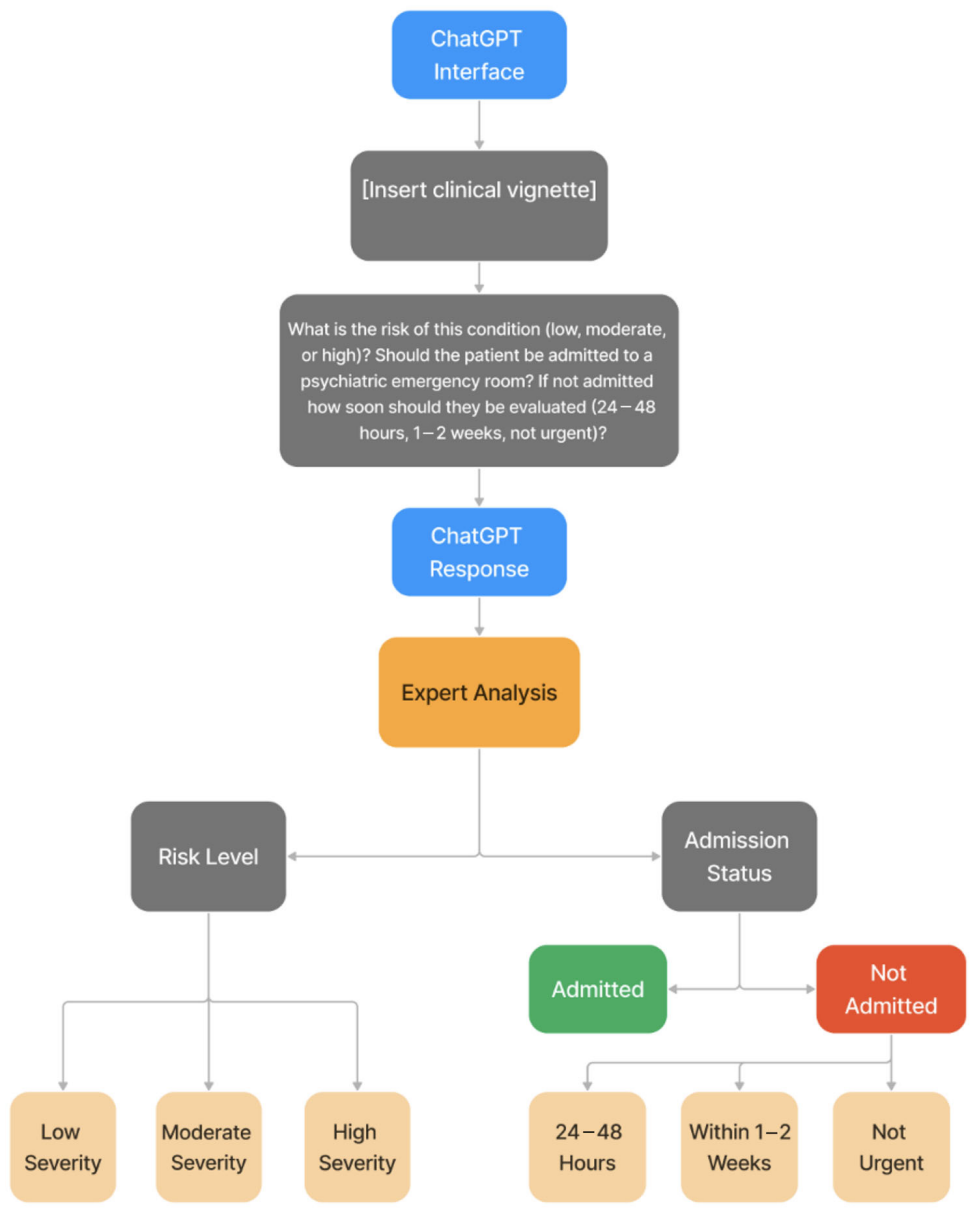
Flowchart depicting the GPT Model responses from clinical vignettes and subsequent expert evaluation based on risk level, admission status, and urgency levels.

The vignettes (see Appendix A) along with the three questions seen in Figure 2 were entered into GPT‐4o, GPT‐4o mini, and GPT‐4 Legacy one time each and the subsequent responses were recorded for data collection into an Excel sheet for a total of 66 responses amongst the different GPT Models (see Appendix B). To ensure response consistency, the responses were generated via three more iterations. A total of 198 additional responses were generated and compared to ensure no significant discrepancies among the responses (Appendix C). The responses from all three models were recorded each time for expert evaluation and Textbox 1 shows a sample response from GPT‐4o. The same vignettes were then evaluated by two nurse practitioners. The nurse practitioners were asked to rate each clinical vignette based on assessment criteria—admission, urgency, and risk—using a three‐point Likert‐type scale. The expert committee, composed of three board‐certified psychiatrists, K.L., K.H., and M.Y., then evaluated the responses by GPT Models qualitatively using a coding framework (see Table 1).

Textbox 1. **Example of generated response by ChatGPT‐4o**

**Vignette:** Emily is a 17‐year‐old Hispanic female high school senior who has recently been experiencing visual and auditory hallucinations. She reports seeing shadows and hearing whispers, particularly when she is alone or trying to fall asleep. Despite these troubling symptoms, Emily continues to perform well academically and is actively involved in her school's drama club. Her parents have observed increased anxiety and some social withdrawal, but Emily has not exhibited aggression or experienced any academic impairment. She denies any thoughts of self‐harm or harm to others. Emily has a history of occasional anxiety and was briefly on sertraline, which she stopped due to mild side‐effects. There is also a family history of anxiety and mild depression.

**Response**: Emily's risk level can be considered **moderate**. Although she is experiencing hallucinations, she continues to function well academically and socially, and she denies any thoughts of self‐harm or harm to others. Her symptoms, though concerning, do not currently suggest an immediate risk of danger to herself or others, which reduces the urgency for psychiatric hospitalization.

Given the presence of hallucinations, increased anxiety, and social withdrawal, it is important that Emily be evaluated by a mental health professional in a timely manner. However, since there is no immediate threat or severe impairment, it is not necessary for her to be admitted to a psychiatric emergency room at this time.

The recommendation would be to have Emily evaluated within **1–2 weeks** by a psychiatrist or psychologist to further assess her condition and to explore potential interventions or treatment options. If her symptoms worsen or if there is any indication of self‐harm, harm to others, or significant functional decline, an emergency evaluation should be sought sooner.

**Table 1:** Rubric for content analysis.

Measures	Question	Description	Scale
Triage Accuracy	Is the answer an accurate reply to the question?	This criterion assesses the accuracy of the risk level, admission status, and overall triage evaluation. “**Accurate**” response fully answers the question with correct information. “**Inaccurate**” indicates the response is incorrect or irrelevant. “**Partially Accurate**” suggests the response is on the right track but either contains some inaccuracies or doesn't fully address all aspects of the question.	Accurate (3), Partially Inaccurate (2), Inaccurate
Clarity	Is the message clearly conveyed?	This measures how easily understandable the response is. “**Yes**” means the response is well‐structured, easy to follow, and free of jargon or ambiguity. “**No**” implies the explanation is confusing, poorly structured, or uses overly complex language. “**Partially**” for partially com response has some clarity but may be improved in certain areas for better understanding.	Yes (3), Partially (2), No (1)
Completeness	Does the response completely answer the question?	This evaluates whether the response fully addresses all elements of the question. “**Complete**” indicates that the response covers all aspects of the question comprehensively. “**Partial**” indicates the response partially addresses the question, but misses some aspects . “**Incomplete**” means the response misses one or more critical elements of the question.	Complete (3), Partial (2), Incomplete (1)
Overall Score	What is the overall score?	This rating reflects the confidence in the response's accuracy, clarity and completeness. “**Low**” suggests effectiveness and appropriateness of the response are likely significantly different from a clinician's judgment of the case vignette. It indicates major issues in accuracy, clarity, inclusivity, completeness, or utility. Or this rating implies that there is a considerable possibility the response's actual effectiveness and appropriateness might differ significantly from what is perceived. While not entirely unreliable, the response has notable deficiencies. “**Moderate**” is used when it is believed that the response is probably close to what is perceived in terms of accuracy, clarity, inclusivity, completeness, and utility. The response is reliable but might have minor areas for improvement. “**High**” indicates a strong level of confidence in the response. It suggests that the true effectiveness and appropriateness of the response are very similar to what is perceived, showing high levels of accuracy, clarity, inclusiveness, completeness, and utility.	High (3), Moderate (2), Low (1)

### Evaluation criteria and measures

The evaluation criteria we created in this study were used to assess the performance of each of the GPT Models and clinicians. The criteria were created based on literature examining the clinical relevance of LLM tools.^35^, ^36^, ^37^ The expert evaluation criteria included triage accuracy, clarity, completeness, and overall score (see Table 1). The expert committee rated responses by GPT Models using the expert evaluation criteria.

We utilized our own triage criteria rather than standardized screening instruments, such as the Columbia Suicide Severity Rating Scale (C‐SSRS),^38^ because our primary goal was to specifically evaluate GPT‐4's ability to replicate clinician judgment during prehospital telephone triage. Standardized instruments, while extensively validated and widely used, primarily focus on assessing particular symptom severity, such as suicide risk, and therefore may not adequately address the broader, context‐driven decision‐making processes involved in real‐time telephone triage. Additionally, our clinical scenarios included diverse psychiatric emergencies, such as suicidal ideation, psychosis, substance use, aggression, and extreme anxiety, which are challenging to evaluate comprehensively with a single standardized scale. By developing tailored triage criteria, we aimed to more accurately reflect the nuanced, holistic decision‐making process that clinicians engage in during psychiatric emergencies.

The authors used a systematic approach to grade each response. All responses were graded on the five criteria on the 3‐point scale, with 3 representing the best fulfillment of that specific criterion (see Table 1). For example, a rating of 3 on triage accuracy would indicate that the GPT Model was able to correctly triage a certain patient fully based on risk level, admission status, and urgency of evaluation (see Figure 2). Conversely, a rating of 1 on completeness would indicate that the response by the GPT Model was incomplete and did not address the requirements of the question.

### Data preparation

Preprocessing of study data included reverse scoring urgency (originally *High* = 1 to *Low* = 3) to match the scale of risk (now *Low* = 1 to *High* = 3) for interpretability. Partial or range (i.e., “1–2” or “2–3”) answers were replaced with the higher value in the range. Risk criteria were mainly assessed using three levels. For responses falling between levels (e.g., “low to moderate” or “moderate to high”), our expert committee adopted a conservative approach by assigning these responses to the higher risk category. Specifically, “low to moderate” responses were classified as “moderate,” and “moderate to high” responses as “high.” This method aligns with clinical best practices to ensure patient safety, appropriate clinical intervention, and reproducibility. Additionally, urgency scores that were originally “0” based on the dependence of admission being “1” were interpreted as “NA” and were not calculated during analysis.

### Analysis selection

We assessed the agreement of clinicians and LLMs using two methodologies. First, we separated them into two groups to determine the Cohen's Kappa (weighted quadratically). Since healthcare providers can vary slightly in their approach to admissions, risk assessment, and urgency by discipline, we used quadratic weights to penalize extreme differences more harshly between clinicians and GPTs. Second, we compared the three models within the GPT group for agreement with Fleiss's Kappa. Descriptive analysis was conducted by calculating the mean (M), standard deviation (SD), median, and interquartile range.

In terms of the Kappa coefficients, ≤0 indicated no agreement, 0.01–0.20 indicated slight agreement, 0.21–0.40 indicated fair agreement, 0.41–0.60 indicated moderate agreement, 0.61–0.80 indicated substantial agreement, and 0.81–1.00 was indicative of nearly perfect agreement.^39^ To interpret agreement between groups, we were guided by similar studies in the literature comparing the performance factors between clinical experts and LLMs.^40^, ^41^

## RESULTS

The GPT Models overall had a strong level of triage accuracy, clarity, completeness, and overall score. Findings are presented using the 3‐point scale evaluated by the psychiatrists (see Table 2). In this study, GPT Models were also compared against each other. Regarding triage accuracy, GPT‐4o had the highest mean score (M = 2.56, SD = 0.68), which was equivalent to GPT‐4 Legacy (M = 2.39, SD = 0.74), and GPT‐4o mini had the lowest mean (M = 2.39, SD = 0.65). Following this, clarity scores were the highest for GPT‐4o (M = 2.65, SD = 0.48), which was also equivalent to GPT‐4 Legacy (M = 2.65, SD = 0.48) and the lowest was GPT‐4o mini (M = 2.36, SD = 0.54). Completeness scores were the highest for GPT‐4 Legacy (M = 2.48, SD = 0.50) followed by GPT‐4o (M = 2.45, SD = 0.56) and lowest for GPT‐4o mini (M = 2.20, SD = 0.66). Overall score was the highest for GPT‐4o (M = 2.53, SD = 0.61) followed by GPT‐4 Legacy (M = 2.47, SD = 0.68) and again the lowest for GPT‐4o mini (M = 2.29, SD = 0.70).

**Table 2:** LLM tools organized by expert evaluation criteria.

Model	Triage accuracy	Clarity	Completeness	Overall
**GPT‐4o**				
M	2.56	2.65	2.45	2.53
SD	0.68	0.48	0.56	0.61
Mdn	3.00	3.00	2.00	3.00
IQR	1.00	1.00	1.00	1.00
**GPT‐4o mini**				
M	2.39	2.36	2.20	2.29
SD	0.65	0.54	0.66	0.70
Mdn	2.00	2.00	2.00	2.00
IQR	1.00	1.00	1.00	1.00
**GPT‐4 Legacy**				
M	2.56	2.65	2.48	2.47
SD	0.74	0.48	0.50	0.68
Mdn	3.00	3.00	2.00	3.00
IQR	1.00	1.00	1.00	1.00

*Note*: Min = 1, Max = 3.

Abbreviations: IQR, interquartile range; M, mean; Mdn, median; SD, standard deviation.

As given in Table 3, the interrater reliability between clinicians and GPT Models was substantial (Cohen's Kappa: admission = 0.77, 95% confidence interval [CI]: 0.70–0.84; risk = 0.78, 95% CI: 0.72–0.85; urgency = 0.76, 95% CI: 0.70–0.83; *p* < 0.001). Among the GPT Models, Kappa values also reflected moderate to substantial agreement (Fleiss’ Kappa: admission = 0.69, risk = 0.63, urgency = 0.72). These Kappa values suggest a high level of concordance, showing substantial agreement across both clinicians and GPT Models. Descriptive analysis showed that the mean scores were 1.73 (SD = 0.45) for admission, 2.12 (SD = 0.83) for risk, and 2.27 (SD = 0.44) for urgency, with minimal variability and consistent scoring patterns across all categories.

**Table 3:** Interrater reliability score between clinicians and GPTs, among GPT Models and descriptive statistics.

Triage measures	Clinicians & GPTs (Cohen's Kappa W^2^)	4o, Mini, Legacy (Fleiss)	95% CI (Cohen's Kappa W^2^)	Mean (SD)	Min–Max
Admission	0.77*	0.69*	0.70–0.84	1.73 (0.45)	1–2
Risk	0.78*	0.63*	0.72–0.85	2.12 (0.83)	1–3
Urgency	0.76*	0.72*	0.70–0.83	2.27 (0.44)	1–3

Abbreviations: CI, confidence interval; SD, standard deviation.

* *p* < 0.001.

**Figure 3:**
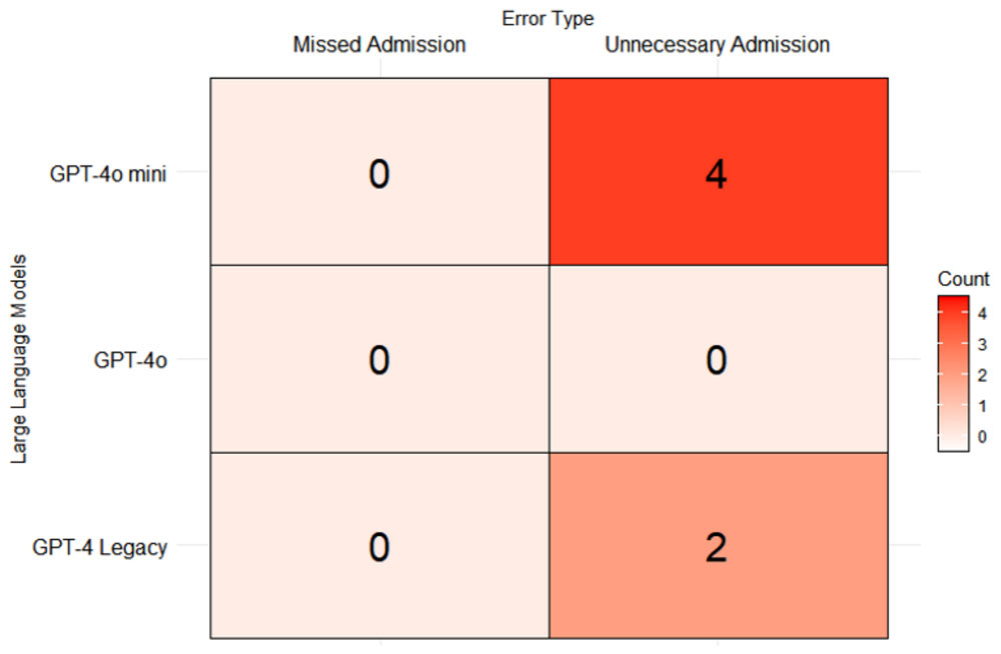
Heat map of false positive and false negative admission errors across GPT Models.

False positives occurred when the GPT Models recommended admission in cases where clinicians did not, resulting in unnecessary admissions. On the other hand, false negatives were when clinicians admitted patients when GPT Models did not, which results in a missed triage patient. We found that there were more total false positives compared to false negatives across GPT Models. In fact, there were no false negatives at all (see Figure 3). The GPT Models in overall responses leaned towards admitting more patients compared to not admitting at all.

To assess response consistency, each clinical vignette was re‐entered into each GPT Model (GPT‐4o, GPT‐4o Mini, and GPT‐4 Legacy) three additional times in separate sessions. Using Fleiss's Kappa across models, there were minimal discrepancies (~3%) observed in admission and urgency (*κ* = 0.48, *p* < 0.32), and significant discrepancies (~24%) observed in risk (*κ* = 0.505, *p* < 0.001). A detailed breakdown of the discrepancy ratios is provided in Appendix C.

## DISCUSSION

This study evaluated the performance of three GPT Models in generating responses to clinical vignettes based on five common categories of psychiatric emergencies experienced by young adults and compared the performance to that of two nurse practitioners. We observed substantial agreement between clinicians and GPT Models as well as significant differences between GPT‐4o mini and the other GPT Models in the following metrics: triage accuracy, clarity, completeness, and overall score.

In terms of triage accuracy, GPT‐4o and GPT‐4 Legacy were both shown to outperform GPT‐4o mini. Previous studies have observed similar results where GPT‐4o mini may compromise accuracy and consistency in responses when answering multiple‐choice exams in different medical specialties.^42^, ^43^ Clarity and completeness were also areas where GPT‐4o mini underperformed in comparison with GPT‐4o and GPT‐4 Legacy, which has also been observed in additional research evaluating the clinical capabilities of both models in managing lumbar disc herniations.^44^ This can be attributed to the fact that it is an LLM tool meant for efficiency and speed for greater accessibility as opposed to consistency in response.^34^ As a result, it is no surprise that GPT‐4o mini had the lowest overall score out of the three GPT Models. While the speed of information retrieval is crucial during crisis calls, these situations often involve sensitive and complex issues that demand clear and thorough responses. By collecting and organizing high‐quality triage information, GPT Models can support clinicians in making well‐informed decisions to improve the overall effectiveness of telemental health triage systems.

Interrater reliability between clinicians and GPT Models was observed with moderate to substantial agreement in admission (*κ* = 0.77), risk (*κ* = 0.78), and urgency (*κ* = 0.76). This interrater reliability indicates that GPT Models can replicate much of the reasoning that clinicians use in decision‐making and can support clinical workflows by offering consistent and reliable assessments for the majority of cases, but discrepancies may arise in edge cases. Since LLMs in this study use probabilistic reasoning as opposed to human intuition, there are certain contextual factors that contribute to variability in clinicians’ decision‐making that LLMs are limited in recognizing.^45^ Suicide risk assessment is an example of a process that relies on understanding contextual factors, such as comorbid conditions and the interplay between mental health and social determinants of health—areas that may exceed LLMs’ algorithms. Calculated risk scores from suicide risk‐assessment tools have a low predictive ability that represents a challenging gray area for GPT Models since they require nuanced judgement beyond direct calculation and merely serve as an aid for clinicians’ decision‐making.^46^

### Comparison of GPT and clinician scores

Compared to clinicians, we noticed that GPT Models on average admitted more patients than necessary. GPT Models’ tendency to over‐admit patients may stem from the model's conservative design, which prioritizes minimizing the risk of adverse outcomes in triage.^47^ This was seen mostly from GPT‐4o mini, which had four unnecessary admissions, and GPT‐4 Legacy, which had two unnecessary admissions. Interestingly, we noticed that GPT‐4o did not over‐admit any patients and we believe this could be due to the fact that the model had been tested by experts on social psychology, bias and fairness, and misinformation introduced by new modalities.^48^ However, compared to clinicians, GPT Models overall appear to overvalue the possibility of less severe mental health crises and recommend immediate referral to the ED, even in cases where less severe presentations could be appropriately managed through outpatient follow‐up or scheduled evaluations over longer periods of time.^49^ The inability to accurately assess levels of risk makes it difficult to consider alternative options. Referrals to community health programs, therapy, or outpatient settings can offer valuable support but without thorough assessment these options are overlooked, creating additional strain for EDs.^50^ The discrepancies shown between the original responses and the three subsequent iterations were minimal; however, it showed how ChatGPT's responses may differ in practice. Iterations across GPT Models deviated from each other in 5% of admissions and were all false positives, highlighting an increasing tendency for ChatGPT to over‐triage compared to clinician assessments. The observed variability, particularly in risk and urgency scoring, suggests potential areas for refinement to enhance clinical accuracy and reliability, ultimately improving patient care and resource allocation.

### Implications

Our findings indicate that GPT Models hold promise as a triage support tool for clinicians confronting psychiatric emergencies. Given the ongoing youth mental health crisis, the shortage of behavioral health professionals, and the need for efficient technology‐supported screening methods, these models can enhance both accessibility and efficiency in patient evaluation.^51^ Although computer‐assisted screening tools already exist for psychiatric disorders in telephone triage, they often overlook real‐time interactions—a crucial factor in prehospital triage where timely feedback is essential to prevent the escalation of emergencies.^52^ Real‐time analysis enables clinicians to quickly implement appropriate interventions. Our comparison of three GPT Models showed that larger models (such as GPT‐4o and GPT‐4 Legacy) provide greater insight and accuracy, yet GPT‐4o mini maintained a clinically acceptable triage performance, suggesting that cost‐effective, smaller models could be particularly beneficial for low‐resource clinics.

Beyond immediate triage, GPT Models also offer broader mental health benefits. They have been shown to function as effective psychoeducation tools by providing actionable steps for mental health concerns.^53^ In addition, these models can reduce stigma through a low‐barrier, conversational interface that encourages individuals who are hesitant to seek help.^54^ They further serve as an intermediary by promoting deeper self‐disclosure,^55^ and they can alleviate administrative burdens by assisting with documentation, clinical hypothesis generation, and the rapid summarization of complex data to help maintain clinical records.^56^

However, implementation of such AI tools presents a number of challenges. It cannot have the “human touch” that clinicians can provide, but can be complementary support that can allow clinicians to focus on delivering empathetic care.^57^ Clinicians require many different competencies during MHTT, but building rapport and communicating effectively with patients are key core competencies of MHTT and overall telephone triage that only a human can provide.^13^ This process can often be difficult and time‐consuming for clinicians coupled with the effect that adolescents are apprehensive to approach mental health services, and have generally negative attitudes toward mental health professionals.^58^ Having an AI assistant can help clinicians conduct risk assessments and perform mental health status examinations, which are core competencies of MHTT that do not involve directly interacting with the patient. GPT Models are not capable of providing accurate and consistent information on their own yet since they frequently “hallucinate” and make reasoning errors,^59^ but they have the potential to help guide clinical examination and compliment clinicians in the future. The integration of clinicians and AI in this way has the potential to significantly enhance the overall MHTT process by combining the strengths of human expertise with the efficiency and support provided by GPT Models.

### Limitations

There are several limitations to this study. First, clinical vignettes can potentially serve as a starting point to identifying GPT Models as a potential triage support tool, but further research will need to incorporate real patient data. Our study focused on a limited range of psychiatric emergency categories and utilized controlled scenarios that do not fully capture the complexity and comorbidity often presented in real‐life patient cases. MHTT is also a relatively novel concept and it is not a standardized protocol across emergency hotlines or crisis lines. Additionally, the evaluation criteria used in this study were extended from previous studies involving the assessment of LLMs and has not been validated. Clinical vignettes are not representative of an interaction between a clinician and a patient, which is much more nuanced and has more variability. The flowchart we used is an extremely simplified version of how patient data could be assessed by GPT Models during a call. The capacity of GPT Models to provide real‐time feedback was also not evaluated since patient information was presented all at once. Our study was also limited to only one LLM although we did compare and contrast the capability of three different versions: GPT‐4o, GPT 4o mini, and GPT‐4 Legacy. GPT Models and other LLMs are constantly evolving and as a result the performance of future LLM models could indicate different findings over time. Future considerations could use other LLMs, such as Cohere, Microsoft Copilot, and Gemini, as well as up‐to‐date GPT Models (GPT‐o1, GPT‐o1 mini).

In our study, we did not have any false negatives. In all cases where clinicians determined that a patient required admission, GPT Models made the same admission decision. This is a notable finding since false negatives represent a critical safety concern. In the context of psychiatric emergencies especially, failing to identify patients who are at risk to themselves or others can lead to adverse outcomes. This raises the question of whether ChatGPT could be helpful as a preliminary mental health screening tool.

### Future considerations

While AI cannot replace professional diagnosis and treatment,^60^ future research can compare ChatGPT's performance against mental health screening instruments, such as the Columbia Suicide Severity Rating Scale (C‐SSRS), the Patient Health Questionnaire‐9 for depression, or the Generalized Anxiety Disorder‐7 Scale for anxiety, rather than focusing solely on clinical decision‐making. The C‐SSRS has been extensively validated as a guide for clinicians to assess suicide risk.^38^, ^61^ Additionally, comparing ChatGPT answers solely to those of clinicians was a limitation because clinician decision‐making can vary due to subjective factors and levels of experience. Measuring ChatGPT's answers against these standardized mental health screening assessments would strengthen the evaluation of its diagnostic ability as well as clarify its ability to be used in a broader setting, such as the general public. In addition, ChatGPT's potential could also be explored by non‐mental‐health professionals, such as primary care physicians and emergency medical personnel. These individuals are often the first point of contact for individuals with mental health concerns but often lack specialized psychiatric training. A tool such as ChatGPT could support decision‐making by being able to provide real‐time, structured guidance in assessing psychiatric symptoms.

## CONCLUSION

Our study contributes to the growing body of literature of AI applications in healthcare by addressing how GPT Models can evaluate patients with acute psychiatric conditions or emergencies. By comparing the performance of these models to that of clinicians, we were able to evaluate the ability of AI to work as a prehospital triage support tool. The findings of this study provide valuable insights for future research on the application of GPT Models in mental health telephone triage and contribute to advancements in AI for mental health care.

## AUTHOR CONTRIBUTIONS

Samanvith Thotapalli conceived the project, collected data, and wrote the manuscript. Musa Yilanli contributed to project conception, assisted with data collection, and organized the study. Emre Sezgin and Daniel I. Jackson analyzed the data and designed the analyses. Ian McKay and William Leever reviewed the clinical vignettes and contributed to the manuscript. Eric Youngstrom supervised the project and provided guidance on the analyses and overall manuscript.

## CONFLICT OF INTEREST STATEMENT

The authors declare no conflicts of interest.

## ETHICS APPROVAL STATEMENT

N/A.

## PATIENT CONSENT STATEMENT

N/A.

## CLINICAL TRIAL REGISTRATION

N/A.

## Supporting information

Supporting information.

## Data Availability

The datasets generated and analyzed during this study are available from the corresponding author upon reasonable request.

## References

[1] Delaney KR . Youth mental health crisis: what's next? J Child Adolesc Psychiatr Nurs. 2024;37:e12480. 39359073 10.1111/jcap.12480

[2] Kemp E , Chen H , Childers C . Mental health engagement: addressing a crisis in young adults. Health Mark Q. 2023;40:153–173. 34825631 10.1080/07359683.2021.2004339

[3] Brunette MF , Erlich MD , Edwards ML , Adler DA , Berlant J , Dixon L , et al. Addressing the increasing mental health distress and mental illness among young adults in the United States. J Nerv Ment Dis. 2023;211:961–967. 38015186 10.1097/NMD.0000000000001734

[4] Holman MS , Williams MN . Suicide risk and protective factors: a network approach. Arch Suicide Res. 2022;26:137–154. 32522102 10.1080/13811118.2020.1774454

[5] Motillon‐Toudic C , Walter M , Séguin M , Carrier JD , Berrouiguet S , Lemey C . Social isolation and suicide risk: literature review and perspectives. Eur Psychiatry. 2022;65:e65. 36216777 10.1192/j.eurpsy.2022.2320 PMC9641655

[6] Hua LL , Lee J , Rahmandar MH , Sigel EJ . Suicide and suicide risk in adolescents. Pediatrics. 2024;153:e2023064800. 38073403 10.1542/peds.2023-064800

[7] Walter HJ , DeMaso DR . Psychiatric emergencies in pediatrics. Pediatr. Care Online. 2022;396486. 10.1542/aap.ppcqr.396486

[8] Saidinejad M , Duffy S , Wallin D , Hoffmann JA , Joseph MM , Schieferle Uhlenbrock J , et al. The management of children and youth with pediatric mental and behavioral health emergencies. Pediatrics. 2023;152:e2023063256. 37581617 10.1016/j.jen.2023.07.005

[9] Williams CYK , Zack T , Miao BY , Sushil M , Wang M , Kornblith AE , et al. Use of a large language model to assess clinical acuity of adults in the emergency department. JAMA Netw Open. 2024;7:e248895. 38713466 10.1001/jamanetworkopen.2024.8895 PMC11077390

[10] Rajab Dizavandi F , Froutan R , Karimi Moonaghi H , Ebadi A , Fayyazi Bordbar MR . Mental health triage from the viewpoint of psychiatric emergency department nurses; a qualitative study. Arch Acad Emerg Med. 2023;11:e70. 38028935 10.22037/aaem.v11i1.2080 PMC10646953

[11] Sands N , Elsom S , Marangu E , Keppich‐Arnold S , Henderson K . Mental health telephone triage: managing psychiatric crisis and emergency. Perspect Psychiatr Care. 2013;49:65–72. 23293999 10.1111/j.1744-6163.2012.00346.x

[12] Matthews S , Cantor JH , Brooks Holliday S , Eberhart NK , Breslau J , Bialas A , et al. Mental health emergency hotlines in the United States: a scoping review (2012–2021). Psychiatr Serv. 2023;74:513–522. 36254453 10.1176/appi.ps.20220128

[13] Sands N , Elsom S , Gerdtz M , Henderson K , Keppich‐Arnold S , Droste N , et al. Identifying the core competencies of mental health telephone triage. J Clin Nurs. 2013;22:3203–3216. 22860919 10.1111/j.1365-2702.2012.04093.x

[14] Michel J , Manns A , Boudersa S , Jaubert C , Dupic L , Vivien B , et al. Clinical decision support system in emergency telephone triage: a scoping review of technical design, implementation and evaluation. Int J Med Inform. 2024;184:105347. 38290244 10.1016/j.ijmedinf.2024.105347

[15] Knebel D , Priglinger S , Scherer N , Klaas J , Siedlecki J , Schworm B . Assessment of ChatGPT in the prehospital management of ophthalmological emergencies—an analysis of 10 fictional case vignettes. Klin Monbl Augenheilkd. 2024;241:675–681. 37890504 10.1055/a-2149-0447

[16] de Koning E , Biersteker TE , Beeres S , Bosch J , Backus BE , Kirchhof CJ , et al. Prehospital triage of patients with acute cardiac complaints: study protocol of HART‐c, a multicentre prospective study. BMJ Open. 2021;11:e041553. 10.1136/bmjopen-2020-041553 PMC7883865 33579765

[17] Raff D , Stewart K , Yang MC , Shang J , Cressman S , Tam R , et al. Improving triage accuracy in prehospital emergency telemedicine: scoping review of machine learning–enhanced approaches. Interact J Med Res. 2024;13:e56729. 39259967 10.2196/56729 PMC11429666

[18] Haque MDR , Rubya S . An overview of chatbot‐based mobile mental health apps: insights from app description and user reviews. JMIR Mhealth Uhealth. 2023;11:e44838. 37213181 10.2196/44838 PMC10242473

[19] Thakkar A , Gupta A , De Sousa A . Artificial intelligence in positive mental health: a narrative review. Front Digit Health. 2024;6:1280235. 38562663 10.3389/fdgth.2024.1280235 PMC10982476

[20] Lee EE , Torous J , De Choudhury M , Depp CA , Graham SA , Kim HC , et al. Artificial intelligence for mental health care: clinical applications, barriers, facilitators, and artificial wisdom. Biol Psychiatry Cogn Neurosci Neuroimaging. 2021;6:856–864. 33571718 10.1016/j.bpsc.2021.02.001 PMC8349367

[21] MacIntyre MR , Cockerill RG , Mirza OF , Appel JM . Ethical considerations for the use of artificial intelligence in medical decision‐making capacity assessments. Psychiatry Res. 2023;328:115466. 37717548 10.1016/j.psychres.2023.115466

[22] Loch AA , Kotov R . Promises and pitfalls of internet search data in mental health: critical review. JMIR Ment Health. 2025;12:e60754. 39964955 10.2196/60754 PMC11855165

[23] Haim M , Arendt F , Scherr S . Abyss or shelter? On the relevance of web search engines’ search results when people google for suicide. Health Commun. 2017;32:253–258. 27196394 10.1080/10410236.2015.1113484

[24] Elyoseph Z , Levkovich I . Beyond human expertise: the promise and limitations of ChatGPT in suicide risk assessment. Front Psychiatry. 2023;14:1213141. 37593450 10.3389/fpsyt.2023.1213141 PMC10427505

[25] Dergaa I , Fekih‐Romdhane F , Hallit S , Loch AA , Glenn JM , Fessi MS , et al. ChatGPT is not ready yet for use in providing mental health assessment and interventions. Front Psychiatry. 2023;14:1277756. 38239905 10.3389/fpsyt.2023.1277756 PMC10794665

[26] Zhang Z , Wang J . Can AI replace psychotherapists? exploring the future of mental health care. Front Psychiatry. 2024;15:1444382. 39544371 10.3389/fpsyt.2024.1444382 PMC11560757

[27] Gutierrez G , Stephenson C , Eadie J , Asadpour K , Alavi N . Examining the role of AI technology in online mental healthcare: opportunities, challenges, and implications, a mixed‐methods review. Front Psychiatry. 2024;15:1356773. 38774435 10.3389/fpsyt.2024.1356773 PMC11106393

[28] Brach W , Košťál K , Ries M . The effectiveness of large language models in transforming unstructured text to standardized formats. Preprint at. 2025; arXiv:2503.02650vX. 10.48550/arXiv.2503.02650

[29] Obradovich N , Khalsa SS , Khan WU , Suh J , Perlis RH , Ajilore O , et al. Opportunities and risks of large language models in psychiatry. NPP Digit Psychiatry Neurosci. 2024;2:8. 39554888 10.1038/s44277-024-00010-z PMC11566298

[30] Malgaroli M , Schultebraucks K , Myrick KJ , Andrade Loch A , Ospina‐Pinillos L , Choudhury T , et al. Large language models for the mental health community: framework for translating code to care. Lancet Digit Health. 2025;7:e282–e285. 39779452 10.1016/S2589-7500(24)00255-3 PMC11949714

[31] OpenAI. GPT‐4. n.d. https://openai.com/index/gpt-4/

[32] OpenAI. GPT‐4 research. 2024. https://openai.com/index/gpt-4-research/

[33] OpenAI. Introducing GPT‐4o and more tools to ChatGPT free users. n.d. https://openai.com/index/gpt-4o-and-more-tools-to-chatgpt-free/

[34] OpenAI. GPT‐4o mini: advancing cost‐efficient intelligence. n.d. https://openai.com/index/gpt-4o-mini-advancing-cost-efficient-intelligence/

[35] Sezgin E , Chekeni F , Lee J , Keim S . Clinical accuracy of large language models and google search responses to postpartum depression questions: cross‐sectional study. J Med Internet Res. 2023;25:e49240. 37695668 10.2196/49240 PMC10520763

[36] Sezgin E , Jackson DI , Kocaballi AB , Bibart M , Zupanec S , Landier W , et al. Can large language models aid caregivers of pediatric cancer patients in information seeking? A cross‐sectional investigation. Cancer Med. 2025;14:e70554. 39776222 10.1002/cam4.70554 PMC11705392

[37] Yilanli M , McKay I , Jackson DI , Sezgin E Large Language Models for Individualized Psychoeducational Tools for Psychosis: a cross‐sectional study. 2024.07.26.24311075. Preprint at medRxiv. 2024. 10.1101/2024.07.26.24311075 PMC13422232 42530475

[38] Bjureberg J , Dahlin M , Carlborg A , Edberg H , Haglund A , Runeson B . Columbia‐Suicide Severity Rating Scale Screen Version: initial screening for suicide risk in a psychiatric emergency department. Psychol Med. 2022;52:3904–3912. 10.1017/S0033291721000751 PMC9811343 33766155

[39] McHugh ML . Interrater reliability: the kappa statistic. Biochem Med. 2012;22:276–282. PMC3900052 23092060

[40] Montiel‐Romero S , Rajme‐López S , Román‐Montes CM , López‐Iñiguez A , Rivera‐Villegas HO , Ochoa‐Hein E , et al. Recommended antibiotic treatment agreement between infectious diseases specialists and ChatGPT®. BMC Infect Dis. 2025;25:38. 39773383 10.1186/s12879-024-10426-9 PMC11706082

[41] Pasli S , Yadigaroğlu M , Kirimli EN , Beşer MF , Unutmaz İ , Ayhan AÖ , et al. ChatGPT‐supported patient triage with voice commands in the emergency department: a prospective multicenter study. Am J Emerg Med. 2025; 94: 0 63–70. 10.1016/j.ajem.2025.04.040 40273640

[42] Jędrzejczak WW , Skarżyński H , Kochanek K . Testing new versions of ChatGPT in terms of physiology and electrophysiology of hearing: improved accuracy but not consistency. 2024. Preprint at medRxiv 10.1101/2024.10.08.24315089

[43] Chang Y , Su C‐Y , Liu Y‐C . Assessing the performance of chatbots on the Taiwan psychiatry licensing examination using the Rasch model. Healthcare. 2024;12:2305. 39595502 10.3390/healthcare12222305 PMC11594248

[44] Wang S , Wang Y , Jiang L , Chang Y , Zhang S , Zhao K , et al. Assessing the Clinical Support Capabilities of ChatGPT‐4o and ChatGPT‐4o Mini in Managing Lumbar Disc Herniation. Preprint at Research Square. 2024. 10.21203/rs.3.rs-5121204/v1 PMC11753088 39844276

[45] Ameen S , Wong M‐C , Yee K‐C , Turner P . AI and clinical decision making: the limitations and risks of computational reductionism in bowel cancer screening. Applied Sciences. 2022;12:3341.

[46] Sequeira L , Strudwick G , Bailey SM , De Luca V , Wiljer D , Strauss J . Factors influencing suicide risk assessment clinical practice: protocol for a scoping review. BMJ Open. 2019;9:e026566. 10.1136/bmjopen-2018-026566 PMC6398626 30782946

[47] Ward M , Maity A , Brown EDL , Cohen A , Schneider D , Ber R , et al. Analysis of ChatGPT in the triage of common spinal complaints. World Neurosurg. 2024;192:e273–e280. 39326666 10.1016/j.wneu.2024.09.086

[48] OpenAI. Hello GPT‐4o. n.d. https://openai.com/index/hello-gpt-4o/

[49] Hugunin J , Davis M , Larkin C , Baek J , Skehan B , Lapane KL . Established outpatient care and follow‐up after acute psychiatric service use among youths and young adults. Psychiatr Serv. 2023;74:2–9. 36223162 10.1176/appi.ps.202200047 PMC9812848

[50] Levin E , Aburub H . Barriers and solutions to comprehensive care for mental health patients in hospital emergency departments. J Ment Health Clin Psychol. 2024;8:26–34.

[51] Lee C , Mohebbi M , O'Callaghan E , Winsberg M . Large language models versus expert clinicians in crisis prediction among telemental health patients: comparative study. JMIR Ment Health. 2024;11:e58129. 38876484 10.2196/58129 PMC11329850

[52] Green S , Sjöström K , Wangel A‐M . Nurses’ perceptions of telephone triage in child and adolescent psychiatric services—an enhanced critical incident technique study. Issues Ment Health Nurs. 2023;44:974–983. 37672771 10.1080/01612840.2023.2237113

[53] Maurya RK , Montesinos S , Bogomaz M , DeDiego AC . Assessing the use of ChatGPT as a psychoeducational tool for mental health practice. Couns. Psychother. Res. 2025;25:e12759.

[54] Lee HS , Wright C , Ferranto J , Buttimer J , Palmer CE , Welchman A , et al. Artificial intelligence conversational agents in mental health: patients see potential, but prefer humans in the loop. Front Psychiatry. 2025;15:1505024. 39957757 10.3389/fpsyt.2024.1505024 PMC11826059

[55] Lee Y‐C , Yamashita N , Huang Y . Designing a chatbot as a mediator for promoting deep self‐disclosure to a real mental health professional. Proc ACM Hum‐Comput Interact. 27 2020;4(31):1–31.

[56] Blease C , Torous J . ChatGPT and mental healthcare: balancing benefits with risks of harms. BMJ Ment Health. 2023;26:e300884. 10.1136/bmjment-2023-300884 PMC10649440 37949485

[57] Rana R , Higgins N, Haque KN, Reilly J, Burke K, Turner K, et al. Feasibility of mental health triage call priority prediction using machine learning. 2024. Preprint at arXiV. 10.48550/ARXIV.2412.00057 PMC11677863 39728664

[58] Radez J , Reardon T , Creswell C , Lawrence PJ , Evdoka‐Burton G , Waite P . Why do children and adolescents (not) seek and access professional help for their mental health problems? a systematic review of quantitative and qualitative studies. Eur Child Adolesc Psychiatry. 2021;30:183–211. 31965309 10.1007/s00787-019-01469-4 PMC7932953

[59] Cheng SW , Chang CW , Chang WJ , Wang HW , Liang CS , Kishimoto T , et al. The now and future of ChatGPT and GPT in psychiatry. Psychiatry Clin Neurosci. 2023;77:592–596. 37612880 10.1111/pcn.13588 PMC10952959

[60] Sezgin E . Artificial intelligence in healthcare: complementing, not replacing, doctors and healthcare providers. Digit Health. 2023;9:20552076231186520. 37426593 10.1177/20552076231186520 PMC10328041

[61] Salvi J . Columbia‐Suicide Severity Rating Scale (C‐SSRS). Emerg Med Pract. 2019;21:CD3–CD4. PMC7974826 31039299

